# Metabolic Profile and Neurogenic Potential of Human Amniotic Fluid Stem Cells From Normal vs. Fetus-Affected Gestations

**DOI:** 10.3389/fcell.2021.700634

**Published:** 2021-07-16

**Authors:** Giedrė Valiulienė, Aistė Zentelytė, Elizabet Beržanskytė, Rūta Navakauskienė

**Affiliations:** Department of Molecular Cell Biology, Institute of Biochemistry, Life Sciences Center, Vilnius University, Vilnius, Lithuania

**Keywords:** mesenchymal stem cells, polyhydramnios, cell differentiation, energy metabolism, neurogenesis

## Abstract

Human amniotic fluid stem cells (hAFSCs) possess some characteristics with mesenchymal stem cells (MSCs) and embryonic stem cells and have a broader differentiation potential compared to MSCs derived from other sources. Although hAFSCs are widely researched, their analysis mainly involves stem cells (SCs) obtained from normal, fetus-unaffected gestations. However, in clinical settings, knowledge about hAFSCs from normal gestations could be poorly translational, as hAFSCs from healthy and fetus-diseased gestations may differ in their differentiation and metabolic potential. Therefore, a more thorough investigation of hAFSCs derived from pathological gestations would provide researchers with the knowledge about the general characteristics of these cells that could be valuable for further scientific investigations and possible future clinical applicability. The goal of this study was to look into the neurogenic and metabolic potential of hAFSCs derived from diseased fetuses, when gestations were concomitant with polyhydramnios and compare them to hAFSCs derived from normal fetuses. Results demonstrated that these cells are similar in gene expression levels of stemness markers (*SOX2*, *NANOG*, *LIN28A*, etc.). However, they differ in expression of CD13, CD73, CD90, and CD105, as flow cytometry analysis revealed higher expression in hAFSCs from unaffected gestations. Furthermore, hAFSCs from “Normal” and “Pathology” groups were different in oxidative phosphorylation rate, as well as level of ATP and reactive oxygen species production. Although the secretion of neurotrophic factors BDNF and VEGF was of comparable degree, as evaluated with enzyme-linked immunosorbent assay (ELISA) test, hAFSCs from normal gestations were found to be more prone to neurogenic differentiation, compared to hAFSCs from polyhydramnios. Furthermore, hAFSCs from polyhydramnios were distinguished by higher secretion of pro-inflammatory cytokine TNFα, which was significantly downregulated in differentiated cells. Overall, these observations show that hAFSCs from pathological gestations with polyhydramnios differ in metabolic and inflammatory status and also possess lower neurogenic potential compared to hAFSCs from normal gestations. Therefore, further *in vitro* and *in vivo* studies are necessary to dissect the potential of hAFSCs from polyhydramnios in stem cell-based therapies. Future studies should also search for strategies that could improve the characteristics of hAFSCs derived from diseased fetuses in order for those cells to be successfully applied for regenerative medicine purposes.

## Introduction

Since the discovery that human amniotic fluid stem cells (hAFSCs) have a broader differentiation potential compared to mesenchymal stem cells (MSCs), obtained from other sources (e.g., bone marrow) (Yan et al., 2013; Bonaventura et al., 2015; Jain et al., 2019), hAFSC therapy has emerged as a new and promising approach in the field of regenerative medicine (Ramasamy et al., 2018). In general, MSCs, especially those obtained from birth-associated or perinatal tissues, are a promising strategy for the treatment of various prenatal and neonatal disorders with complex multifactorial etiologies, such as bronchopulmonary dysplasia, congenital heart disease, intraventricular hemorrhage, and even type III spinal muscular atrophy (Nitkin et al., 2020; Ahn et al., 2021; Shaw et al., 2021).

In addition, hAFSCs show only minimal replicative senescence (Alessio et al., 2018; Gasiūnienė et al., 2020), which makes amniotic fluid an especially attractive stem cell source. Considering the fact that amniotic fluid may be obtained *via* routine prenatal diagnostics, with minimal invasiveness and minimal ethical issues, this source of stem cells is of particular importance for the treatment of newborns. This is especially relevant considering the cases of polyhydramnios, when excessive accumulation of amniotic fluid occurs and amniocentesis (amniotic fluid reduction) is inevitable (Kleine et al., 2016). In such cases, hAFSCs could be proliferated and modulated *ex vivo* to be further used for autologous stem cell therapy.

According to literature, polyhydramnios complicates between 0.5 and 2% of all pregnancies. Several possible causes may lead to polyhydramnios, such as maternal diabetes, rhesus isoimmunization, fetal chromosome abnormalities (e.g., Down syndrome and Edward’s syndrome), and malformations of the gastrointestinal tract (e.g., fetal gastric atony and esophageal atresia) or the central nervous system (Yefet and Daniel-Spiegel, 2016). Sadly, in the case of polyhydramnios, abnormalities of the central nervous system, such as neural tube defects (e.g., *spina bifida occulta*, meningocele, and myelomeningocele), are the most common cause of malformation (Kouamé et al., 2013). Recently, amniotic fluid stem cell therapy *in utero* emerged as a promising strategy for myelomeningocele (MMC) treatment (Abe et al., 2019). The aforementioned study showed that in the retinoic acid-induced rat MMC model, treatment with hAFSCs reduced neuronal damage and induced neuro-regeneration in a hepatocyte growth factor-dependent manner. In addition, studies demonstrated that hAFSCs may suppress neuronal inflammation and restore neuronal cells; therefore, in the future, hAFSCs could possibly be applied for the treatment of perinatal neurological diseases (Abe et al., 2021).

Although promising, the field of hAFSC cellular therapy for treating neonatal birth defects is still in its infancy. Studies that have evaluated hAFSC applicability to treat neural tube defects (Abe et al., 2019) examined the applicability of hAFSCs obtained *via* amniocentesis from the pregnancies without fetal abnormalities. For preclinical studies of other conditions such as congenital heart disease, respiratory tract anomalies, and perinatal gastrointestinal disorders, hAFSCs from healthy gestations were also used (Kunisaki, 2018). However, in real clinical settings when autologous hAFSC transplantation would be desirable, the knowledge obtained while investigating hAFSCs from healthy gestations may be poorly translational, as hAFSCs from normal and fetus-affected gestations could possibly differ in their metabolic status and consequently in their differentiation and trophic potential. It should be emphasized that the aforementioned potential may also be affected by the gestational age at which hAFSCs are obtained, as some studies showed that hAFSCs obtained from early second trimester of gestation are more potent compared to hAFSCs obtained at a later gestational age (Shaw et al., 2017). However, in the case of polyhydramnios, hAFSCs may be acquired only when the polyhydramnios condition occurs (in most cases, polyhydramnios develops late in the second or in the third trimester of pregnancy) (Ursachen and Therapie, 2013). It should also be emphasized that scientific data about hAFSCs derived from fetus-affected gestations are very limited. Our group previously showed that hAFSCs from normal and fetus-affected gestations had similar stem cell characteristics and potential to differentiate toward mesodermal (adipogenic, osteogenic, chondrogenic, and myogenic) as well as ectodermal (neurogenic) lineages (Gasiūnienė et al., 2019b; Zentelytė et al., 2020). However, no scientific data exist on the metabolic characteristics of these two types of hAFSCs, nor are there any data on the neurogenic and neurotrophic potential of hAFSCs obtained exclusively from polyhydramnios samples. Therefore, it is crucial to describe the basic characteristics and determine the potential of hAFSCs derived from fetus-diseased gestations concomitant with polyhydramnios in order to explore their future therapeutic applicability in averting highly debilitating pregnancy complications.

Consequently, the main objective of this study was to evaluate the general metabolic and inflammatory characteristics, as well as the neurogenic and neurotrophic potential of hAFSCs, obtained from fetus-affected gestations with polyhydramnios, in comparison with normal fetus-unaffected gestations. Therefore, we examined the cellular energy flux of tested hAFSCs and assessed their total ATP production, as well as the mitochondrial membrane potential and quantities of intracellular reactive oxygen species (ROS). For neurogenic induction, several different chemical cocktails were used, and their effect on neurite formation and the gene expression of the main neural differentiation-associated factors (e.g., *NCAM1-2*, *NSE*, *NES*, *TUBB3*, *NEUROD1*, *SYP*, *MAP2*, *BDNF*, and *NGF*) were evaluated. In addition, expression of intracellular neural proteins (Nestin, Musashi, Lin28a and β-tubulin) and secretion of certain trophic and inflammatory factors (BDNF, VEGF, TNFα, and IL-6) were also examined.

On the whole, the results of our study expand the knowledge about the general properties and differences of hAFSCs, obtained from the third-trimester fetus-affected gestations concomitant with polyhydramnios in comparison to hAFSCs derived from the second-trimester normal gestations. Data show that tested hAFSCs from pathological and normal gestations differ in their phenotypic and inflammatory characteristics, as well as neurogenic potential. Therefore, future studies should address further functional investigations, as well as the modulation of hAFSCs from pathological gestations obtained from polyhydramnios samples *ex vivo*, in order for these cells to be successfully used for autologous stem cell therapies in treating neonatal conditions.

## Materials and Methods

### Isolation of hAFSCs

Amniotic fluid samples were obtained by amniocentesis from mid-second-trimester pregnancies (16–17 weeks of gestation; patient age: 39 ± 2.1 years) from healthy women who needed prenatal diagnostics but in whom no genetic abnormalities were detected and from specimens of polyhydramnios, when amnioreduction was needed at the third trimester of gestation (32 weeks; patient age: 27.7 ± 7.4 years). No aneuploidies were detected during clinical diagnostics in samples from both groups. hAFSCs {*n* = 3 samples from healthy gestations and *n* = 3 samples from pathological gestations concomitant with polyhydramnios ([Pat1: diagnosed with fetal gastric atony], [Pat2: diagnosed with fetal esophageal atresia], [Pat3: diagnosed with Treacher Collins syndrome])} were isolated using a two-stage protocol as described in Gasiūnienė et al. (2019b) (protocol approved by the Vilnius Regional Biomedical Research Ethics Committee, No. 158200-18/7-1049-550, version No. 1). Briefly, samples were centrifuged at 500 × *g* for 20 min, supernatant was removed, and cell pellets were washed once with PBS. After centrifugation, cells were resuspended in complete growth medium consisting of DMEM with 10% FBS (Gibco, Thermo Fisher Scientific), 100 U/ml penicillin, and 100 μg/ml streptomycin (Gennaxxon bioscience) and plated in culture flasks. First, cell colonies (mostly of epithelial morphology, first stage) appeared after 10–15 days, and the non-adhering cells were collected and transferred to new culture flasks. After the appearance of cell colonies (hAFSCs, second stage), growth medium was changed every 3 days.

All patients involved in this study signed a written consent. The personal patient data were encrypted before handing over to researchers.

### Cultivation of Isolated hAFSCs

Isolated hAFSCs were maintained in complete growth medium and subcultured at approximately 80% confluence using 0.05% trypsin-EDTA solution (Gennaxxon bioscience), and the cells were reseeded at a density of 1 × 10^4^ cells/cm^2^. All experiments were performed at five to seven passages. During cultivation, the cell population doubling time (in days) was calculated using the following formula: DT = time(days) × log(2)/log(c2/c1), where c1 is the number of seeded cells and c2 is the number of collected cells (Roth, 2006).^1^

### Flow Cytometry Analysis

Human amniotic fluid stem cells were characterized by surface marker expression. Cells were collected and washed twice with phosphate buffered saline (PBS) with 1% bovine serum albumin (BSA). A total of 6 × 10^4^ cells/sample were resuspended in 50 μl of 1% BSA/PBS and incubated with mouse anti-human antibodies for CD9, CD13, CD15, CD31, CD34, CD44, CD56, CD73, CD90, CD105, CD117, CD133, CD146, CD166, CD309, CD338, HLA-ABC, and HLA-DR. Cells were incubated with antibodies in the dark at +4°C for 30 min, washed twice with 1% BSA/PBS, and then analyzed. For intracellular flow cytometry staining, cells were washed with PBS, fixed with 2% paraformaldehyde at RT for 10 min, and then permeabilized using 0.1% Triton X-100 in PBS/1% BSA solution at RT for 15 min. After centrifugation, cells were resuspended in PBS/BSA/Triton X-100 solution and incubated for 30 min at 4°C in the dark with mouse anti-human or rabbit anti-human antibodies for Nestin, Musashi 1, LIN28a, and TUBB3. Goat anti-mouse or goat anti-rabbit IgG (H + L) Highly Cross-Absorbed Alexa Fluor^®^ 488 (Invitrogen) conjugated secondary antibodies were used to label Nestin, MSI1, and LIN28a for another 30 min at 4°C in the dark (fluorophore and manufacturer information is listed in Supplementary Table 1). After incubation, cells were washed twice with PBS/1% BSA and analyzed. The measurements were carried out using a Millipore Guava^®^ easyCyte 8HT flow cytometer, using the InCyte 2.2.2 software. Ten thousand events were collected for each sample.

### Differentiation Assay

Neurogenic differentiation of hAFSCs was induced using four different chemical cocktails (Table 1). Cells were seeded to culture dishes, and when the confluency reached 60%, growth media was removed, cell monolayer was washed with PBS, and pre-induction (for protocols I, II, and III) or differentiation (protocol IV) media was applied. After 24 h of pre-induction (for protocols I, II, and III), cells were washed with PBS and differentiation media was applied. hAFSCs were further differentiated for 72 h. Neural differentiation was assessed by neurite formation and a total of >1,000 cells were examined in each sample. Neurite length was measured with NIH ImageJ using the NeuronJ plugin.

**TABLE 1:** Neurogenic differentiation conditions.

Protocol identification number	Preinduction (24 h)	Differentiation media (72 h)
I	DMEM (low glucose) 10% heat-inactivated FBS	BrainPhys^TM^, 100 U/ml penicillin and 100 μg/ml streptomycin, 1% NeuroCult^TM^, 1 mM 8-Bromo-cAMP, 0.3 mM IBMX, 5 mM KCl, 2 μM RA
II	100 U/ml penicillin 100 μg/ml streptomycin	BrainPhys^TM^, 100 U/ml penicillin and 100 μg/ml streptomycin, 1% NeuroCult^TM^, 50 ng/ml BDNF, 100 ng/ml NGF, 5 mM KCl, 2 μM RA
III	20 ng/ml FGF 20 ng/ml EGF	BrainPhys^TM^, 100 U/ml penicillin and 100 μg/ml streptomycin, 1% NeuroCult^TM^, 1 mM 8-Bromo-cAMP, 0.3 mM IBMX, 50 ng/ml BDNF, 100 ng/ml NGF, 5 mM KCl, 2 μM RA
IV	-	DMEM/F12, 100 U/ml penicillin and 100 μg/ml streptomycin, 1% N2 Supplement, 2 μM RA

### Immunofluorescence Analysis

For immunofluorescence, hAFSCs were seeded in Lab-Tek Chamber slides (Thermo Fisher Scientific), cultivated as control, or differentiated (with III protocol) toward neurogenic lineage and then fixed with 4% formaldehyde for 15 min at RT, washed with PBS, and then permeabilized with 10% Triton X-100/PBS for 20 min at RT. After washing with PBS, cells were blocked using 1% BSA/10% goat serum/PBS for 30 min at 37°C. For detection of NCAM1, cells were incubated with primary mouse antibodies against NCAM1 (15 μg/ml) (Abcam) for 1 h at 37°C, followed by incubation with secondary goat anti-mouse IgG (H + L) Highly Cross-Adsorbed, Alexa Fluor^®^ 594 antibodies (1:400) (Invitrogen) for 1 h at 37°C. For detection of TUBB3 and Vimentin, cells were incubated with FITC-conjugated rabbit anti-beta III tubulin antibodies (1:100) (Abcam) or Alexa Fluor^®^ 488-conjugated rabbit anti-Vimentin antibodies (1:150) (Abcam) for 1 h at 37°C. F-actin was detected using Alexa Fluor^®^ 594 Phalloidin (Thermo Fisher Scientific) for 30 min at RT. Cells were washed several times with 1% BSA/PBS after each incubation. Nuclei were stained using 300 nM DAPI solution (Invitrogen) for 10 min at RT and slides were mounted with Dako Fluorescent Mounting Medium (Agilent Technologies). Labeled cells were analyzed using a Zeiss Axio Observer (Zeiss) fluorescent microscope, 63 × objective magnification with immersion oil and Zen BLUE software.

### RNA Isolation and RT-qPCR

Total RNA from hAFSCs was isolated using TRIzol^®^ reagent (Applied Biosystems) as recommended by the manufacturer. For the gene expression analysis, cDNA synthesis was performed using SensiFAST^TM^ cDNA Synthesis Kit (Bioline). RT-qPCR was performed with SensiFAST^TM^ SYBR^®^ No-ROX Kit (Bioline) on the Rotor-Gene 6000 thermocycler with Rotor-Gene 6000 series software (Corbett Life Science). *GAPDH* and *RPL13A* genes (geometric mean of their Ct values) were used for normalization of the mRNA and the relative gene expression was calculated using the ΔΔCt method (compared to undifferentiated control). The list of primers (Metabion International AG) is provided in Supplementary Table 2.

### BDNF, VEGF, TNFα, IL-1β, IL-6, and IL-10 Quantification in Conditioned Media

Enzyme-linked immunosorbent assay (ELISA) was used to determine the secreted levels of BDNF, VEGF, TNFα, IL-1β, IL-6, and IL-10 in conditioned media of control and differentiated hAFSCs. For this purpose, cells were seeded in culture flasks and were cultivated for 3 days as control (untreated) cells or cells induced to differentiate toward neurogenic lineage using II differentiation protocol. Then, control and differentiated hAFSCs were washed thoroughly with PBS and cell media was changed to NutriStem^®^ hPSC XF medium (Biological Industries) for 3 days, after which both the cells and the media were collected separately. All ELISA detection kits were purchased from R&D Systems and all procedures were carried out according to the manufacturer’s instructions and plates were read with spectrophotometer Infinite M200 Pro (Tecan). For a blank control, NutriStem^®^ hPSC XF medium was used. hAFSC protein lysates were obtained using RIPA buffer (150 mM NaCl, 10 mM EDTA, pH 8.0, 10 mM Tris, pH 7.4, 0.1% SDS, 1% deoxycholate, and 1% NP-40 in PBS, pH 7.6). Cell protein concentrations and conditioned media concentrations were measured with Infinite M200 Pro using DC Protein Assay (BioRad Laboratories) according to the manufacturer’s instructions. BDNF, VEGF, TNFα, and IL-6 values as well as protein yield in conditioned media were normalized to the total amount of cell protein.

### Energetic Profile Analysis

Metabolic activity of hAFSCs was measured using Abcam Extracellular O_2_ Consumption Assay (ab197243), TMRE-Mitochondrial Membrane Potential Assay (ab113852), Glycolysis Assay (ab197244), and Luminescent ATP Detection Assay (ab113849). All procedures were carried out following the manufacturer’s instructions. Shortly, for Extracellular O_2_ Consumption Assay, 5 × 10^5^ of trypsinized cells were resuspended in 150 μl of fresh media and transferred to a 96-well plate, and 10 μl of Extracellular O_2_ Consumption Reagent was added to each well. A few drops of mineral oil were added to cover the well and prevent evaporation. The plate was read using a Varioskan Flash multimode reader (Thermo Fisher Scientific) at 37°C for 3 h at 2-min intervals at Ex/Em = 380/650 nm. For TMRE-Mitochondrial Membrane Potential Assay, 1 × 10^5^ of hAFSCs were resuspended in PBS/0.2% BSA and incubated with 400 nM TMRE for 30 min at 37°C. Samples were analyzed with a Millipore Guava^®^ easyCyte 8HT flow cytometer, using the InCyte 2.2.2 software. Ten thousand events were collected for each sample. For Glycolysis Assay, 5 × 10^5^ of trypsinized cells per sample were washed with Respiration Buffer and transferred to a 96-well plate. Ten microliters of Glycolysis Assay Reagent was added to each sample well, and the plate was read with a Varioskan Flash multimode reader at 37°C for 3 h at 1.5-min intervals at Ex/Em = 380/615 nm. For ATP Detection Assay, 5 × 10^4^ cells were seeded into a white 96-well plate; the next day, 50 μl of detergent was added into each well and the plate was placed on a shaker for 5 min at 600–700 rpm. Then, 50 μl of Substrate Solution was added to each well and the plate was again placed on a shaker for 5 min at 600–700 rpm. The plate was dark adapted by covering it for 10 min and then luminescence was measured by using a Varioskan Flash multimode reader.

### Evaluation of ROS Levels

Cellular ROS of hAFSCs were determined using the DCFDA Cellular ROS Detection Assay Kit (ab113851, Abcam) and all procedures were carried out according to the manufacturer’s instructions. In brief, 2.5 × 10^4^ cells were incubated with 25 μM of 2′,7′-dichlorofluorescin diacetate (DCFDA) for 30 min at 37°C and then analyzed using a Millipore Guava^®^ easyCyte 8HT flow cytometer with InCyte 2.2.2 software. Ten thousand events were collected for each sample. For fluorescence microscopy, 1.5 × 10^4^ cells per well were seeded in a 48-well plate and incubated with 25 μM DCFDA for 45 min at 37°C and then observed using an EVOS FL microscope (Thermo Fisher Scientific).

### Statistical Analysis

All experiments were performed in triplicate; data were expressed as the mean ± SD. Statistical analysis was conducted using Student’s *t*-test and one-way ANOVA with Tukey’s *post hoc* test in GraphPad Prism software.

## Results

### Characterization of Human AFSCs From Fetus-Unaffected and Fetus-Affected Gestations

**FIGURE 1:**
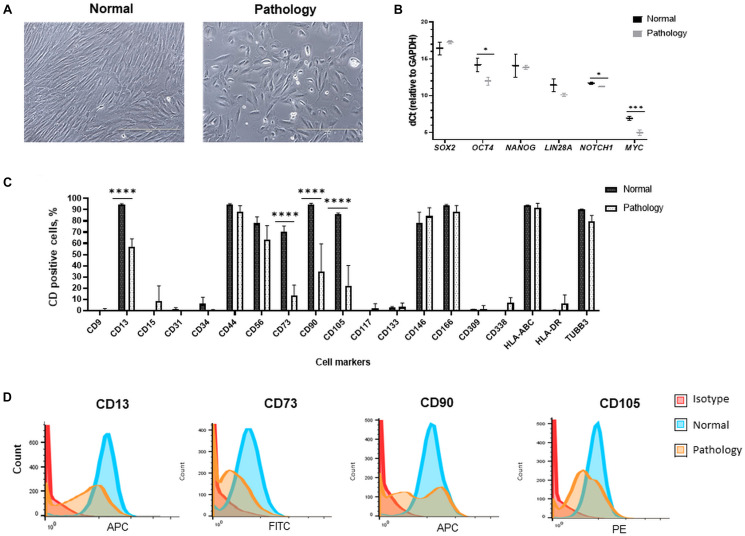
Morphological, gene expression, and cell surface marker characterization of hAFSCs derived from normal and fetus-affected gestations. **(A)** Morphology of hAFSCs cells from a healthy donor (Normal) and pathological gestation (Pathology [Pat1]) amniotic fluid samples (representative images; scale bar = 400 μm). **(B)** RT-qPCR analysis of pluripotency gene markers in samples of hAFSCs of normal (Normal, *n* = 3) and fetus-pathological (Pathology, *n* = 3) gestations. mRNA expression levels were normalized to *GAPDH* and presented as mean values of ΔCt ± SD. *Denotes significant difference with *p* < 0.05 and ***denotes significant difference with *p* < 0.005, as evaluated using two sample *t*-test. **(C)** Cell surface marker expression of CD9, CD13, CD15, CD31, CD34, CD44, CD56, CD73, CD90, CD105, CD117, CD133, CD146, CD166, CD309, CD338, HLA-ABC, and HLA-DR and cell expression of TUBB3 determined in hAFSCs from healthy and fetus-affected gestations by flow cytometry analysis. Data shown as percentage (*n* = 3) and values are indicated as mean ± SD. ****Denotes significant difference with *p* < 0.0001, as evaluated using one-way ANOVA with Tukey’s *post hoc* test. **(D)** The immunophenotypic characteristics of representative samples of hAFSCs of a healthy (Normal) gestation and hAFSCs from polyhydramnios (Pathology, [Pat1]) samples, determined by flow cytometric analysis after incubation with fluorescent-conjugated antibodies against cell surface antigens CD13, CD73, CD90, and CD105.

In this study, human amniotic fluid-derived stem cells, obtained from the amniotic fluid of the second-trimester fetus-unaffected pregnancies (in figures denoted as “Normal”), as well as cells obtained *via* amnioreduction procedures from the third trimester of fetus-affected gestations (in figures denoted as “Pathology”) were used. Stem cells from normal gestations had typical elongated spindle-shaped morphology, whereas hAFSCs obtained from polyhydramnios were distinguished by their round-shaped appearance (Figure 1A). Cells from fetus-unaffected and fetus-affected gestations also differed by their doubling time in early passages (p1–p5): as hAFSCs doubling time was 2.20 ± 0.45 days for the “Normal” group and 2.67 ± 0.47 days for the “Pathology” group, differences were not statistically significant [calculated according to Roth, 2006 (see text footnote 1); data not shown].

In addition, differences in expression of mesenchymal cell surface markers were also observed between studied groups: expression of CD13 (membrane alanyl aminopeptidase), CD73 (5′-nucleotidase), CD90 (thymocyte differentiation antigen 1), and CD105 (endoglin) were significantly more pronounced (up to 96%, *p* < 0.0001) in hAFSCs from fetus-unaffected gestations (Figures 1C,D). However, expression of CD44 (homing cell adhesion molecule), CD56 (neural cell adhesion molecule 1, NCAM1), CD146 (melanoma cell adhesion molecule), and CD166 (activated leukocyte cell adhesion molecule) mesenchymal cell surface markers were of comparable magnitude (Figure 1C). Both studied groups of hAFSCs had low expression (less than 10%) of hematopoietic and endothelial cell marker CD15 (Lewis X antigen), CD31 (platelet endothelial cell adhesion molecule), CD34 (hematopoietic progenitor cell antigen), CD133 (prominin-1), and CD309 (fetal liver kinase 1), as well as negligible expression of other stem cell markers, such as CD9 (tetraspin) and CD117 (proto-oncogene c-kit). It should be noticed that both hAFSCs from fetus-unaffected gestations and fetus-affected gestations were highly positive for β-tubulin protein expression (Figure 1C), which is known to be a marker of neural cells.

Furthermore, RT-qPCR gene expression analysis revealed that hAFSCs from fetus-unaffected gestations and hAFSCs from fetus-affected gestations are positive for pluripotency gene markers, such as *SOX2*, *OCT4*, *NANOG*, *LIN28A*, *NOTCH1*, and *MYC* (Figure 1B). Interestingly, evidently higher expression of *OCT4* (up to 4.6-fold, *p* < 0.05) and *MYC* (up to 3.8-fold, *p* < 0.005) were observed in hAFSCs from polyhydramnios specimens.

### Assessment of Metabolic Differences Between hAFSCs From Fetus-Affected and Fetus-Unaffected Gestations

**FIGURE 2:**
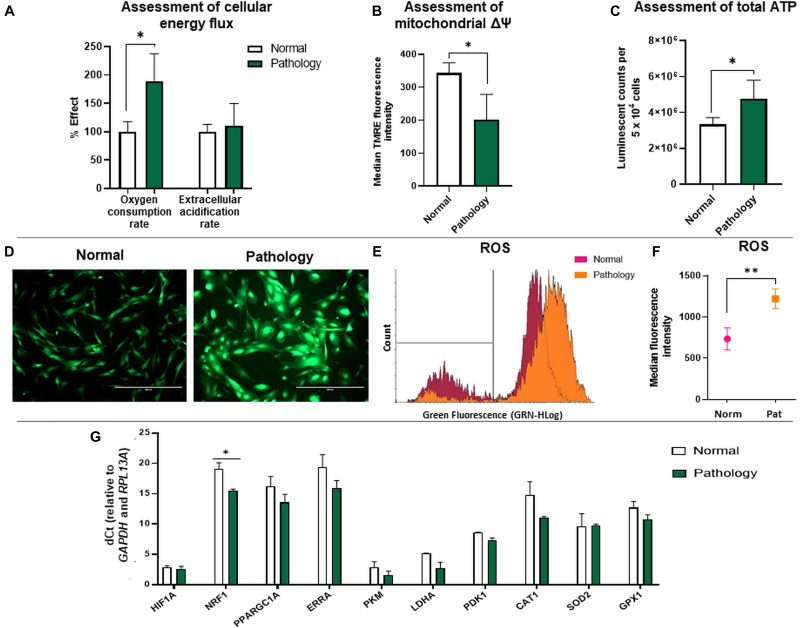
Cellular bioenergetics analysis of hAFSCs from normal and fetus-affected gestations. **(A)** Assessment of cellular energy flux for hAFSCs, obtained from fetus-unaffected (Normal, *n* = 3) and fetus-affected (Pathology, *n* = 3) gestations, shown as a percentage relative to hAFSCs from fetus-unaffected gestations. Comparative measurements were taken using Abcam Extracellular oxygen consumption assay (ab197243) and Abcam Glycolysis assay (ab197244). **(B)** Analysis of mitochondrial membrane potential (Δψm) of hAFSCs, obtained from fetus-unaffected (Normal, *n* = 3) and fetus-affected (Pathology, *n* = 3) gestations; values are indicated as mean ± SD. Analysis was performed using Abcam TMRE Mitochondrial membrane potential assay kit (ab113852). **(C)** Assessment of cellular energy content (ATP) of hAFSCs, obtained from fetus-unaffected (Normal, *n* = 3) and fetus-affected (Pathology, *n* = 3) gestations. Measurements were performed using Abcam Luminescent ATP detection assay kit (ab113849). **(D–F)** ROS production measurement in hAFSCs, obtained from fetus-unaffected (Normal, *n* = 3) and fetus-affected (Pathology, *n* = 3) gestations, performed using Abcam DCFDA Cellular ROS detection assay kit (ab113851). Qualitative evaluation with fluorescence microscopy **(D)**: representative images of ROS production in Normal and Pathology groups (scale bar = 400 μm). Quantitative evaluation with flow cytometry **(E,F)** representative images of ROS production **(E)** and median fluorescence intensity evaluation **(F)** in Normal and Pathology [Pat2] groups. **(G)** RT-qPCR analysis of genes related to cell metabolism and respiration: *HIF1A*, *NRF1*, *PPARGC1A*, *ERRA*, *PKM*, *LDHA*, *PDK1, CAT1, SOD2*, and *GPX1* in control hAFSCs from normal and fetus-affected gestations. mRNA expression levels were normalized to *GAPDH* and *RPL13A* and presented as mean values of ΔCt ± SD. *Denotes significant difference with *p* < 0.05, as evaluated using Student’s *t*-test.

The metabolic potential of hAFSCs obtained from fetus-unaffected gestations, as well as hAFSCs obtained *via* amnioreduction procedures from the fetus-affected gestations, was determined. Firstly, oxygen consumption rate was evaluated and compared between the study groups. Analysis revealed statistically significant (*p* < 0.05) differences between hAFSCs from normal and fetus-affected gestations, as hAFSCs obtained from polyhydramnios samples were respiring more efficiently (oxygen consumption rate was approximately two-fold higher in the “Pathology” group in comparison to the “Normal” group; Figure 2A). However, extracellular acidification rate (Figure 2A), which resembles the intensity of glycolysis, was of comparable degree in both “Normal” and “Pathology” groups of hAFSCs.

Secondly, we compared the relative mitochondrial membrane potential values between the study groups using the TMRE accumulation test. Obtained data showed that TMRE median fluorescence intensity was approximately 1.69-fold higher (*p* < 0.05) in hAFSCs from fetus-unaffected gestations compared to hAFSCs from polyhydramnios samples (Figure 2B). In accordance with glycolysis and oxidative phosphorylation analysis results, assessment of total ATP content confirmed that hAFSCs from polyhydramnios are more energetic, as their ATP content, measured by the luminescent counts per 5 × 10^4^ cells, was approximately 1.4-fold higher (*p* < 0.05) compared to hAFSCs from fetus-unaffected gestations (Figure 2C).

Because of the variance in mitochondrial oxidative phosphorylation rate and mitochondrial membrane potential values among hAFSCs obtained from fetus-unaffected vs. fetus-affected gestations, we performed more thorough investigation of additional parameters, associated with cellular bioenergetics. Estimation of ROS production revealed that hAFSCs obtained from polyhydramnios generate higher levels of ROS, which was demonstrated by increased median fluorescence intensity of 2′,7′-dichlorofluorescein (DCF) [highly fluorescent DCF is formed upon oxidation of 2′,7′-dichlorofluorescein diacetate (DCFDA) by ROS]. In hAFSCs from polyhydramnios, DCF signal was 1.66-fold higher (*p* = 0.054) in comparison to hAFSCs from normal gestations (Figures 2D–F).

In addition, gene expression analysis (Figure 2G) revealed that hAFSCs obtained from polyhydramnios samples can be characterized by higher expression of the *NRF1* (nuclear respiratory factor 1) gene, which acts as a transcription factor regulating some key metabolic genes required for respiration (Yuan et al., 2018). However, no significant differences between groups were detected regarding gene expression of other genes related to cell metabolism and respiration: *HIF1A* (hypoxia-inducible factor 1-alpha), *PPARGC1A* (peroxisome proliferator-activated receptor gamma coactivator 1 alpha), *ERRA* (estrogen related receptor alpha), *PKM* (pyruvate kinase), *LDHA* (lactate dehydrogenase A), and *PDK1* (pyruvate dehydrogenase kinase 1), as well as genes involved in antioxidant defense: *CAT1* (catalase 1), *SOD2* (superoxide dismutase 2), or *GPX1* (glutathione peroxidase 1).

### Morphological Changes of hAFSCs From Fetus-Affected vs. Fetus-Unaffected Gestations Upon Neurogenic Induction

**FIGURE 3:**
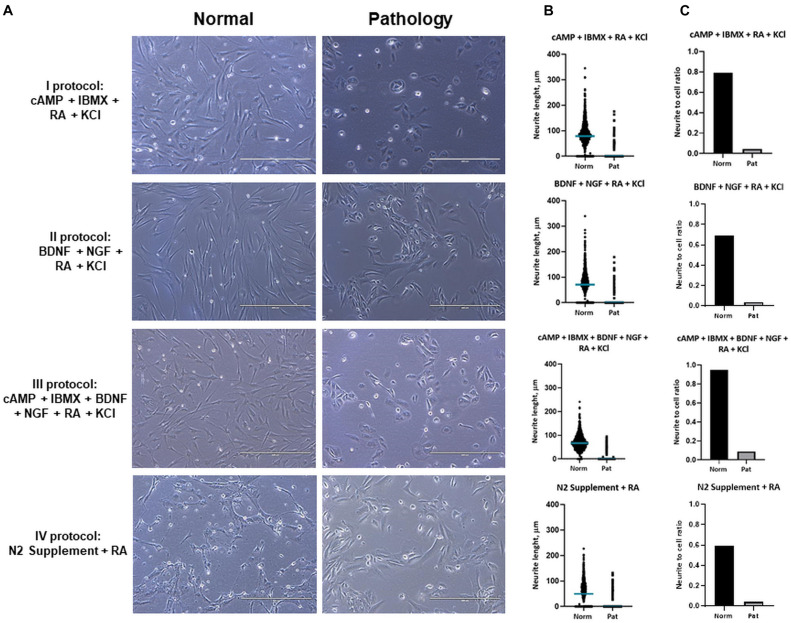
Neuronal differentiation of hAFSCs from normal and fetus-affected gestations. **(A)** Representative images of hAFSCs from normal (Normal) and fetus-pathological (Pathology [Pat1]) gestations upon 72-h treatments with different neuronal differentiation inducing cell culture medias, denoted as I–IV protocols (see Table 1 in section “Materials and Methods”; scale bars = 400 μm). **(B)** Neurite length (μm) estimation for neuro-differentiated hAFSCs, obtained from fetus-unaffected (Normal, *n* = 3) and fetus-affected (Pathology, *n* = 3) gestations was performed using ImageJ program with the NeuronJ plugin; all estimated values (*n* > 1,000) were presented in scatter plot with median value denoted. **(C)** Neurite number per cell for neuro-differentiated hAFSCs, obtained from fetus-unaffected (Normal, *n* = 3) and fetus-affected (Pathology, *n* = 3) gestations was estimated and presented as neurite-to-cell ratio (*n* > 1,000).

Results of differentiation analysis demonstrated that hAFSCs from fetus-unaffected gestations were more susceptible to neurogenic differentiation induction (Figure 3). All four neurogenic differentiation induction strategies (chemical cocktail Nos. I–IV) after 72-h treatment induced visible morphological changes in hAFSCs, obtained from fetus-unaffected gestations (“Normal” group). However, morphological alterations in hAFSCs from the “Pathology” group upon neurogenic differentiation induction was only modest (Figure 3A). In order to quantitatively evaluate the neurogenic differentiation in hAFSCs from fetus-affected vs. fetus-unaffected gestations, neurite length (Figure 3B) and neurite-to-cell ratio (Figure 3C) were counted. Results indicated that hAFSCs from normal gestations generated the longest neurites (median value: 78.695 μm) upon treatment with cAMP + IBMX + RA + KCl (induction cocktail No. I). However, the highest neurite-to-cell ratio (0.95/1) was observed in hAFSCs from normal gestations, when treated with the combination cAMP + IBMX + BDNF + NGF + RA + KCl (induction cocktail No. III).

**FIGURE 4:**
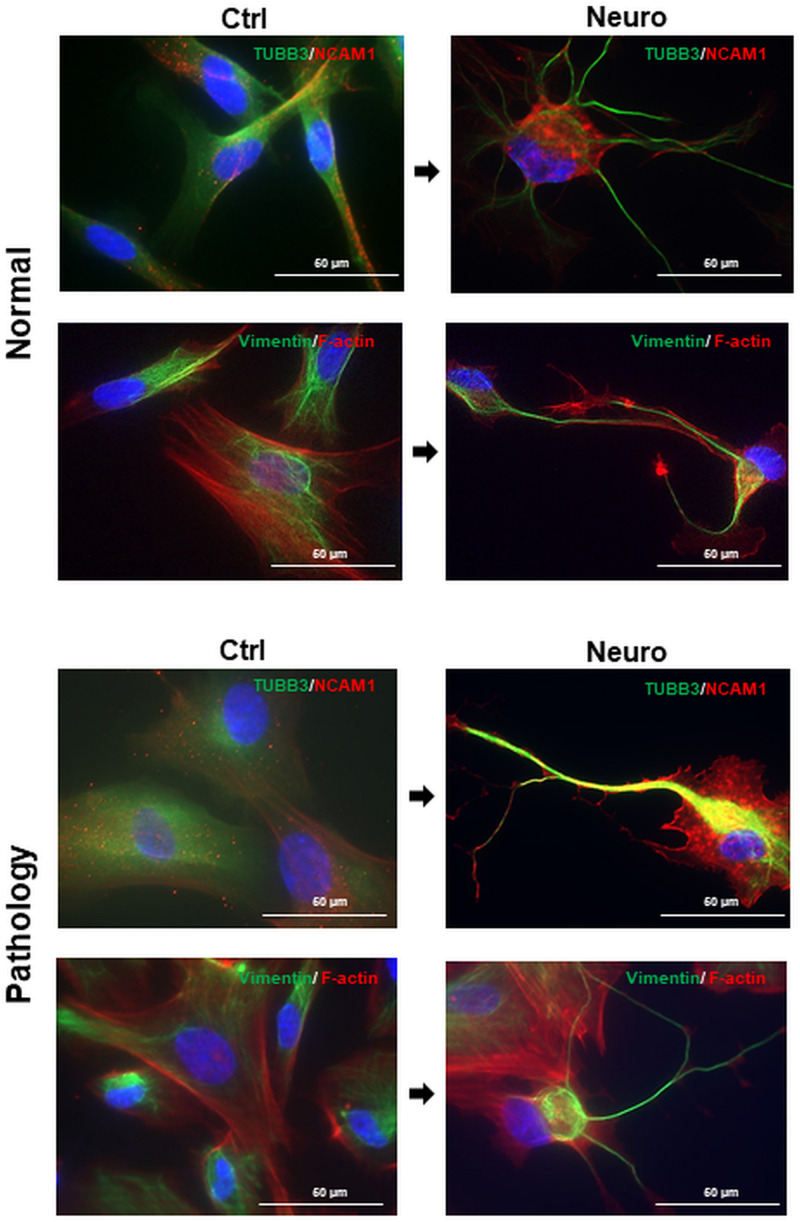
Cytoskeletal reorganization and neural marker expression of control and neural differentiation-induced hAFSCs derived from normal and fetus-affected gestations. Representative images of healthy hAFSCs (Normal) and fetus-affected gestations (Pathology [Pat3]) shown. Neural differentiation was induced after 24-h exposure to pre-induction media, enriched with 20 ng/ml FGF and 20 ng/ml EGF, and further 3-h treatment with neuronal differentiation-inducing cell culture media, supplemented with 1 mM 8-Bromo-cAMP, 0.3 mM IBMX, 50 ng/ml BDNF, 100 ng/ml NGF, 5 mM KCl, and 2 μM RA (see III protocol in section “Materials and Methods,” Table 1). Immunofluorescence analysis of hAFSCs showing positive cells for neural marker TUBB3 and cytoskeletal protein Vimentin (green) as well as neural marker NCAM1 and cytoskeletal protein F-actin (red). Nuclei were counterstained with DAPI (blue); scale bar = 50 μm.

Furthermore, we noticed that upon treatment with the combination of cAMP + IBMX + BDNF + NGF + RA + KCl, both “Normal” and “Pathology” group hAFSCs acquired neural-like morphology very fast—even after 3-h treatment, neural-like cells in hAFSC cultures could be detected (Figure 4). Immunofluorescence analysis of these neuro-induced cells revealed drastic reorganization of hAFSC cytoskeleton (realignment of β-tubulin, vimentin, and F-actin), as well as redistribution of neural marker NCAM1 (Figure 4).

### Evaluation of Neural Gene Expression in Neuro-Induced hAFSCs From Fetus-Affected vs. Fetus-Unaffected Gestations

The RT-qPCR technique was used to evaluate the effect of neural differentiation induction protocols on hAFSC gene expression of neural progenitor cell markers (*SOX2*, *NES*, *NEUROD1*, *VIM*, and *TUBB3*), markers of differentiated post-mitotic neural cells (*NSE, NCAM1-2*, *GAD1*, *TPH1*, *TPH2, MAP2*, and *SYP*), glial markers (*GFAP* and *S100B*), oligodendrocyte precursor cell markers (*PDGFRA*), and genes of (neuro)trophic factors (*BDNF*, *NGF*, *NTF3*, *NTF4, VEGFA, TGFB1*, and *HBEGF*), as well as expression of (neuro)trophic factor receptors (*NTRK1*, *NTRK2*, *NTRK3*, and *FGFR1*).

**FIGURE 5:**
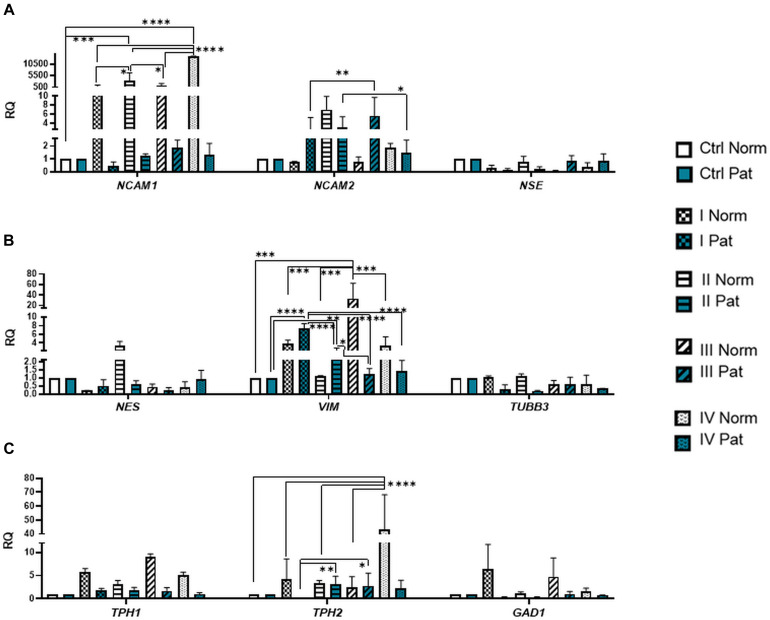
Gene expression profile of neuronal markers, neurotransmitter-producing enzymes, and cytoskeletal proteins in hAFSCs from normal and fetus-affected gestations induced to neuronal differentiation. RT-qPCR analysis of hAFSCs **(A)** neuronal markers *NCAM1*, *NCAM2*, and *NSE*; **(B)** cytoskeletal protein genes *NES*, *VIM*, and *TUBB3*; and **(C)** neurotransmitter-producing enzyme genes *TPH1*, *TPH2*, and *GAD1*. Gene expression analysis was performed using control hAFSCs (not treated, Ctrl) and neuronal differentiation-induced hAFSCs (treated for 72 h with I–IV differentiation protocols; see Table 1 in section “Materials and Methods”) from normal (Norm, *n* = 3) and fetus-pathological (Pat, *n* = 3) gestations. RT-qPCR data are represented as relative fold change over undifferentiated control, normalized for the housekeeping genes *GAPDH* and *RPL13A*; values are indicated as mean ± SD. *Denotes significant difference with *p* < 0.05, **denotes significant difference with *p* < 0.01, ***denotes significant difference with *p* < 0.005, and ****denotes significant difference with *p* < 0.0001, as evaluated using one-way ANOVA with Tukey’s *post hoc* test.

**FIGURE 6:**
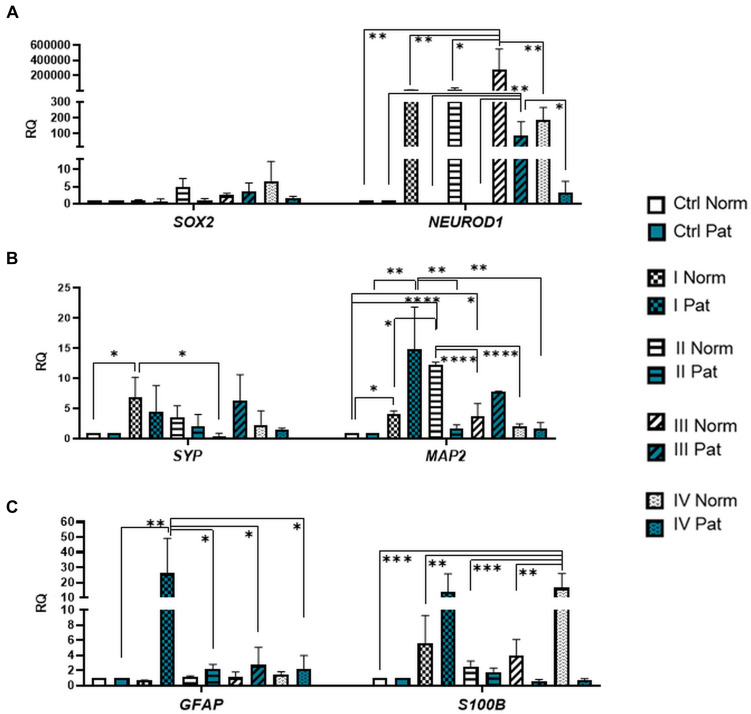
Gene expression profile of neuronal transcription factors and post-mitotic neuronal and glial markers in hAFSCs from normal and fetus–affected gestations induced to neuronal differentiation. RT-qPCR analysis of **(A)** hAFSCs neuronal transcription factors *SOX2* and *NEUROD1*, **(B)** markers of differentiated post-mitotic neural cells *SYP* and *MAP2*, and **(C)** glial markers *GFAP* and *S100B*. Gene expression analysis was performed using control hAFSCs (not treated, Ctrl) and neuronal differentiation-induced hAFSCs (treated for 72 h with I–IV differentiation protocols; see Table 1 in section “Materials and Methods”) from normal (Norm, *n* = 3) and fetus-pathological (Pat, *n* = 3) gestations. RT-qPCR data are represented as relative fold change over undifferentiated control, normalized for the housekeeping genes *GAPDH* and *RPL13A*; values are indicated as mean ± SD. *Denotes significant difference with *p* < 0.05, **denotes significant difference with *p* < 0.01, ***denotes significant difference with *p* < 0.005, and ****denotes significant difference with *p* < 0.0001, as evaluated using one-way ANOVA with Tukey’s *post hoc* test.

**FIGURE 7:**
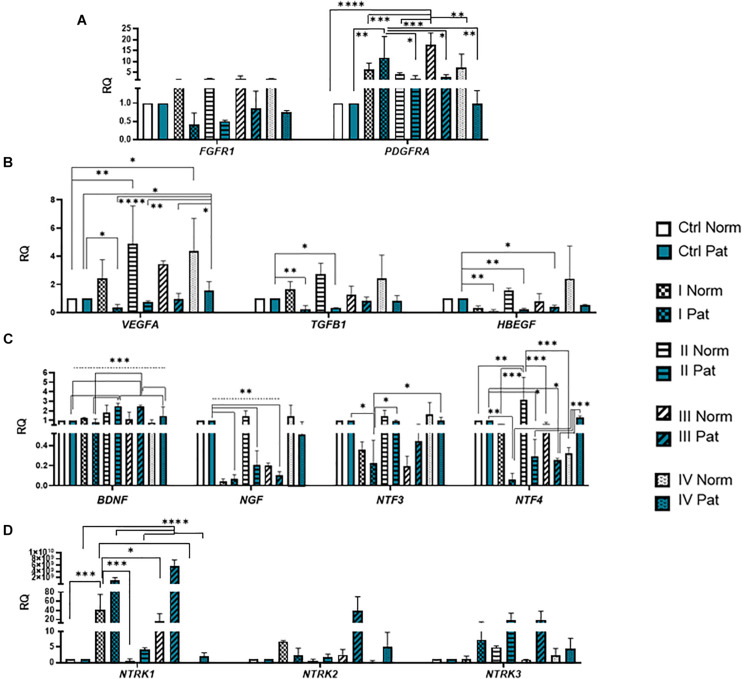
Gene expression profile of growth and neurotrophic factors in hAFSCs from normal and fetus–affected gestations induced to neuronal differentiation. RT-qPCR analysis of **(A)** growth factor receptors *FGFR1* and *PDGFRA*, **(B)** growth factors *VEGFA, TGFB1*, and *HBEGF*, as well as **(C)** neurotrophic factors *BDNF*, *NGF*, *NTF3*, and *NTF4* and **(D)** their receptors *NTRK1*, *NTRK2*, and *NTRK3*. Gene expression analysis was performed using control hAFSCs (not treated, Ctrl) and neuronal differentiation-induced hAFSCs (treated for 72 h with I–IV differentiation protocols; see Table 1 in section “Materials and Methods”) from normal (Norm, *n* = 3) and fetus-pathological (Pat, *n* = 3) gestations. RT-qPCR data are represented as relative fold change over undifferentiated control, normalized for the housekeeping genes *GAPDH* and *RPL13A*; values are indicated as mean ± SD. *Denotes significant difference with *p* < 0.05, **denotes significant difference with *p* < 0.01, ***denotes significant difference with *p* < 0.005, and ****denotes significant difference with *p* < 0.0001, as evaluated using one-way ANOVA with Tukey’s *post hoc* test.

Results revealed that upon 72-h neural differentiation induction in hAFSCs, obtained from fetus-unaffected gestations, *NCAM1* gene expression was profoundly upregulated, whereas in hAFSCs from polyhydramnios, changes in *NCAM1* gene expression were negligible (Figure 5A). In hAFSCs from normal gestations, the strongest *NCAM1* expression (increase by 3,376-fold, *p* < 0.0001) was induced when using the combination N2 Supplement + RA (induction cocktail No. IV). However, the strongest effect on *NCAM2* and *NES* gene expression (approximately sevenfold and threefold, respectively; NS) was observed upon treatment with a combination of BDNF + NGF + RA + KCl (induction cocktail No. II) (Figures 5A,B). In addition, treatment with BDNF + NGF + RA + KCl had the greatest impact on gene expression of transcription factor *SOX2* (approximately 5-fold; NS; Figure 6A), as well as on expression of the neuron-specific cytoskeletal protein *MAP2* gene (approximately 12-fold, *p* < 0.0001; Figure 6B). Furthermore, in hAFSCs obtained from fetus-unaffected gestations, 72-h treatment with BDNF + NGF + RA + KCl enhanced gene expression of neurotrophic factors *BDNF* and *NTF4* (Figure 7C), though increase was moderate (accordingly: 1.8- and 3.2-fold; NS). This treatment also upregulated gene expression of other trophic factors (Figure 7B), such as *VEGFA* (up to 5-fold, *p* = 0.001) and expression of cytokine *TGFB1* (up to 2.7-fold; NS).

We have also evaluated the effect that neural differentiation-inducing treatments have on gene expression of neurotransmitter-producing enzyme genes, such as tryptophan hydroxylase 1 and 2 (*TPH1* and *TPH2*) and the gene of glutamate decarboxylase1 (*GAD1*). Gene expression of *TPH1* and *GAD1* was induced in hAFSCs from normal gestations after treatment with cAMP + IBMX + RA + KCl and after treatment with cAMP + IBMX + BDNF + NGF + RA + KCl (approximately by 5- to 9-fold, NS), whereas *TPH2* gene expression was upregulated the most strongly upon treatment with N2 Supplement + RA (approximately 44-fold, *p* < 0.0001; Figure 5C). In addition, all neural differentiation induction schemes used profoundly increased gene expression of key neuronal differentiation transcription factor *NEUROD1* (Figure 6A). However, the greatest increase in *NEUROD1* gene expression (up to 277,473-fold, *p* < 0.01) was observed after combined treatment with cAMP + IBMX + BDNF + NGF + RA + KCl. In hAFSCs from normal gestations, this treatment scheme also had the strongest effect on cytoskeletal protein Vimentin gene expression (increase up to 33-fold, *p* < 0.005; Figure 5B) and the receptor of PDGF gene expression (increase up to 17.6-fold, *p* < 0.0001; Figure 7A).

As it is evident from the gene expression data, hAFSCs obtained from polyhydramnios samples were much less inducible to neural differentiation, compared to hAFSCs from normal gestations. However, gene expression of glial marker *GFAP* (Figure 6C; “Pat” vs. “Norm” when treated with protocol I differed 39-fold, *p* = 0.13), as well as expression of neurotrophic receptor genes, was upregulated more intensely in hAFSCs from fetus-affected gestations [“Pat” vs. “Norm when treated with protocol III differed in the *NTRK1* gene expression approximately 335,000-fold (*p* < 0.01), *NTRK*2 15.5-fold (*p* = 0.096), *NTRK3* 21-fold (*p* = 0.251); Figure 7D].

**FIGURE 8:**
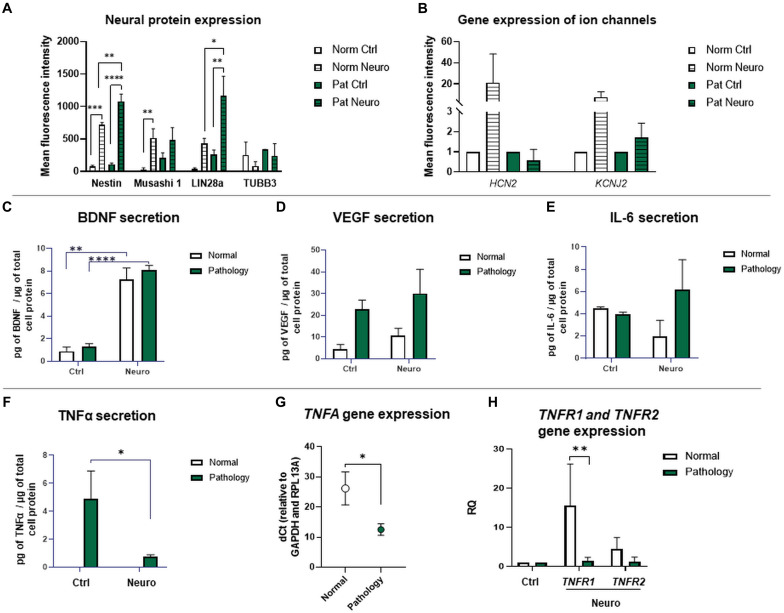
Gene expression of ion channels and protein expression of neuro-induced hAFSCs from fetus-affected vs. fetus-unaffected gestations. **(A)** Expression analysis of neuronal differentiation-associated proteins Nestin, Musashi 1, LIN28a, and TUBB3. Protein expression was evaluated flow cytometrically, estimating the mean fluorescence intensity. Values are indicated as mean ± SD (*n* = 3). Neural differentiation was induced after 24-h exposure to pre-induction media, enriched with 20 ng/ml FGF and 20 ng/ml EGF, and further 72-h treatment with neuronal differentiation-inducing cell culture media, supplemented with 50 ng/ml BDNF, 100 ng/ml NGF, 5 mM KCl, and 2 μM RA (see II protocol in section “Materials and Methods,” Table 1). **(B)** RT-qPCR analysis of *HCN2* (Hyperpolarization activated cyclic nucleotide gated potassium and sodium channel) and *KCNJ2* (Potassium inwardly rectifying channel subfamily member 2) neural ion channel gene expression. Gene expression analysis was performed using control hAFSCs (not treated, Ctrl) and neuronal differentiation-induced hAFSCs (differentiated for 72 h using II protocol, see Table 1 in section “Materials and Methods”) from normal (Norm, *n* = 3) and fetus-pathological (Pat, *n* = 3) gestations. RT-qPCR data are represented as relative fold change over undifferentiated control, normalized for the housekeeping genes GAPDH and RPL13A; values are indicated as mean ± SD. **(C–F)** Secretion levels of differentiation-associated proteins BDNF **(C)**, VEGF **(D)**, and of pro-inflammatory cytokines IL-6 **(E)** and TNFα **(F)** in undifferentiated or differentiated toward neurogenic lineage (differentiated for 72 h using II protocol, see Table 1 in section “Materials and Methods”) hAFSCs from healthy (Normal, *n* = 3) and fetus-affected (Pathology, *n* = 3) gestations. Secretion of proteins was assessed by using ELISA kits from R&D Systems. **(G)** RT-qPCR analysis of *TNFA* gene in undifferentiated hAFSCs from healthy (Normal, *n* = 3) and fetus-affected (Pathology, *n* = 3) gestations. mRNA expression levels were normalized to *GAPDH* and *RPL13A* and presented as mean values of ΔCt ± SD. **(H)** RT-qPCR analysis of TNFα receptor genes *TNFR1* and *TNFR2* in undifferentiated or differentiated toward neurogenic lineage (differentiated for 72 h using II protocol, see Table 1 in section “Materials and Methods”) hAFSCs from healthy (Normal, *n* = 3) and fetus-affected (Pathology, *n* = 3) gestations. mRNA expression levels were normalized to *GAPDH* and *RPL13A* and presented as fold change over undifferentiated control. *Denotes significant difference with *p* < 0.05, **denotes significant difference with *p* < 0.01, and ****denotes significant difference with *p* < 0.0001, as evaluated using Student’s *t*-test.

Considering the fact that the most effective in neural marker induction was the chemical cocktail consisting of BDNF + NGF + RA + KCl, we additionally tested its effect on neural ion channel gene expression (Figure 8B). RT-qPCR analysis revealed that upon neurogenic induction, gene expression of *HCN2* (Hyperpolarization activated cyclic nucleotide gated potassium and sodium channel) and *KCNJ2* (Potassium inwardly rectifying channel subfamily member 2) ion channels was more strongly upregulated in hAFSCs from healthy gestations (37-fold and 4.3-fold respectively, although differences were not statistically significant). Consequently, these results support our observation that hAFSCs from healthy gestations are more susceptible to neural differentiation in comparison to hAFSCs from fetus-affected gestations with polyhydramnios.

### Protein Expression Analysis of Neuro-Induced hAFSCs From Fetus-Affected vs. Fetus-Unaffected Gestations

Expression of neuronal differentiation-associated proteins Nestin, Musashi 1, LIN28a, and neuronal-specific TUBB3 was evaluated flow cytometrically by measuring the mean fluorescence intensity in control and 72-h differentiated hAFSCs from normal and pathological gestations (Figure 8A). For hAFSCs, neurogenic induction chemical cocktail consisting of 50 ng/ml BDNF + 100 ng/ml NGF + 2 μM RA + 5 mM KCl (II protocol) was used. Data of protein expression analysis demonstrated that protein expression of intermediate filament Nestin was significantly upregulated upon neurogenic differentiation induction in both hAFSCs from healthy gestations and gestations with polyhydramnios (*p* < 0.005), though a greater increase was observed in hAFSCs from polyhydramnios samples (*p* < 0.01). Protein expression of stem cell and neural progenitor marker Musashi 1 was also activated by neurogenic induction media. However, only in hAFSCs from normal gestations was this augmentation statistically significant (*p* < 0.01). Similarly to the pattern of protein expression of Nestin, expression of transcription factor LIN28a was also upregulated more profoundly in neuro-differentiated hAFSCs from polyhydramnios samples (*p* < 0.05).

In order to evaluate the secretion of BDNF, VEGF, IL-1β, IL-6, IL-10, and TNFα by untreated and neuro-differentiated hAFSCs, obtained from normal and fetus-affected gestations, we performed ELISA-based analysis and compared secretion of aforementioned trophic and pro-inflammatory factors between study groups. In this case, for neurogenic differentiation induction, the same treatment with 50 ng/ml BDNF, 100 ng/ml NGF, 5 mM KCl, and 2 μM RA (protocol No. II) has been chosen. The yield of secretome fractions was determined based on protein enrichment, as evaluated by BCA assay. The resulting values of micrograms of secreted proteins per microgram of total cell protein were as follows: “Normal” control 75.39 ± 0.18, “Pathology” control 64.33 ± 3.86, “Normal” differentiation 61.72 ± 2.4, and “Pathology” differentiation 95.52 ± 30.14.

The results of our study revealed that secretion of neurotrophic factor BDNF was significantly increased after 72 h of neurogenic differentiation induction in both hAFSCs from the “Normal” and “Pathology” groups to 8.3-fold (*p* < 0.01) and 6.2-fold (*p* < 0.0001), respectively (Figure 8C). In addition, in both cell types, in hAFSCs from normal gestations and gestations with polyhydramnios, secretion of trophic factor VEGF was also increased upon neurogenic differentiation induction approximately by 2.2- and 1.3-fold, respectively (Figure 8D). It is worth to mention that before neurogenic differentiation induction, both BDNF and VEGF secretion was higher in hAFSCs, obtained from gestations concomitant with polyhydramnios (Figures 8C,D).

Interestingly, ELISA analysis demonstrated that studied hAFSCs, obtained from different sources, do differ in pro-inflammatory cytokine TNFα secretion—hAFSCs from the “Pathology” group secreted approximately 4.9 pg of TNFα/μg of total cell protein, whereas no secretion of TNFα was detected in hAFSCs from normal gestations (Figure 8F). It should be emphasized that after 72 h of neurogenic differentiation induction, TNFα secretion by hAFSCs from polyhydramnios was reduced by 6.6-fold (*p* < 0.05). Furthermore, we have evaluated the gene expression of *TNFA* and its receptors *TNFR1* and *TNFR2*. RT-qPCR analysis revealed that control, untreated, hAFSCs from healthy gestations have lower *TNFA* expression (dCt value higher by 13.7 points, which means approximately 13,170-fold difference; *p* < 0.05) compared to control hAFSCs from polyhydramnios (Figure 8G). Though gene expression of TNFα receptor gene *TNFR2* was of comparable degree between control hAFSCs from “Normal” and “Pathology” groups, *TNFR1* gene expression was significantly upregulated in hAFSCs from normal gestations after neuronal differentiation induction (*p* < 0.01; Figure 8H).

In addition, we also evaluated hAFSC secretion of other inflammation-associated cytokines IL-1β, IL-6, and IL-10. ELISA analysis revealed that hAFSCs, either from healthy or pathological gestations, do not secrete pro-inflammatory cytokine IL-1β. Secretion of anti-inflammatory cytokine IL-10 was also not detected (data not shown). However, secretion analysis of IL-6 indicated that both types of tested hAFSCs (groups “Norm” and “Pat”) were comparable in the level of secretion of this pro-inflammatory cytokine (Figure 8E). Although subtle changes in IL-6 secretion were observed upon hAFSC neurogenic induction (in hAFSCs from healthy gestations, secretion was downregulated, while in hAFSCs from polyhydramnios, it was slightly upregulated), observed differences were not statistically significant.

Collectively, these data show that despite having a lower neurogenic potential, when evaluated morphologically (Figure 3), hAFSCs from gestations with polyhydramnios do hold similar or even greater capacity of producing neural proteins intracellularly. They also have the ability to secrete neurotrophic factors extracellularly, such as BDNF and VEGF, in comparable amounts to hAFSCs from healthy gestations. However, it should also be mentioned that hAFSCs from pathological gestations with polyhydramnios do exhibit different inflammatory phenotypes in comparison to hAFSCs from normal pregnancies, as secretion of pro-inflammatory cytokine TNFα was detected only from hAFSCs, obtained from polyhydramnios samples.

## Discussion

Amniotic fluid-derived stem cells are considered as a new and potential experimental approach toward improving various conditions, such as autoimmune diseases (Yang et al., 2021), ischemic heart disease (Fang et al., 2021), and Alzheimer’s disease (Gatti et al., 2020). They are also suggested to help to suppress cancer (Jafari et al., 2021) or even improve spermatogenesis (Mobarak et al., 2021). However, the main interest goes to treating congenital anomalies such as *spina bifida*, congenital diaphragmatic hernia, and congenital heart disease (Di Bernardo et al., 2014; Kunisaki, 2018; Abe et al., 2019). Although advances in the medical field provide some level of treatment for patients who have been prenatally diagnosed with these anomalies, they continue to burden pediatric care and account for a significant part of infant mortality, morbidity, and hospitalization days worldwide.

The use of hAFSCs for therapeutic applications presents both logical and practical choice for a number of reasons. Stem cell harvesting can be performed prior to delivery by amniocentesis, during which a small sample of amniotic fluid is aspirated from the amniotic sac. Since amniocentesis is a routine tool for diagnostic purposes (sampling amniotic cells for aneuploidies and genetic abnormalities), it is considered as a safe procedure performed with needle aspiration under ultrasound guidance (Daum et al., 2019). Amniotic fluid is an easily accessible source rich with potent cells and even small volumes of sample can produce an abundant quantity of amniocytes and amniotic fluid stem cells (Gasiūnienė et al., 2020). On the contrary, isolating stem cells from other prenatal tissues such as chorionic villi, umbilical cord blood (cordocentesis), fetal skin, liver, or muscle is more technically challenging (Cadrin and Golbus, 1993; Cheng, 2018). In addition, hAFSCs have a robust proliferation rate that exceeds MSCs from other sources when expanded under identical conditions (Kunisaki et al., 2007). Therefore, once the prenatal diagnosis is complete, hAFSCs can be easily propagated *in vitro* for therapeutic purposes. Furthermore, amniotic fluid stem cells originate from the fetus, thus enabling autologous therapeutic applications without concern for immunological rejection upon delivery either prenatally or during the postnatal period (Abe et al., 2021).

While there is a potential for hAFSCs to be a tool in regenerative medicine-based approaches, there is a relative shortage of information on disease-specific amniotic fluid stem cells. A major portion of conducted studies investigated only the benefits of hAFSCs isolated from healthy pregnancies. The results of these studies could cause some limitations on the application of stem cells derived from fetus-affected gestations to be used in clinical settings due to possible key differences in stem cell characteristics between stem cells of these two sources (healthy and fetus-affected gestations). It should be emphasized that there is no scientific data on metabolic characteristics of hAFSCs obtained from fetus-diseased gestations, nor are there any data on the neurogenic and neurotrophic potential of hAFSCs obtained, for example, from polyhydramnios samples. Therefore, it is crucial to describe the general characteristics and determine the potential of hAFSCs derived from fetus-diseased gestations concomitant with polyhydramnios in order to test their future therapeutic applicability in treating highly devastating pregnancy complications. Previous studies of our research group demonstrated that undifferentiated hAFSCs from normal gestations may produce ATP either *via* a tricarboxylic acid cycle or anaerobic glycolysis (both pathways almost equally active) (Gasiūnienė et al., 2019a). However, there were still no scientific data on the respiratory potential and metabolic activity of hAFSCs obtained from polyhydramnios. Increasing knowledge supports the idea that the status of stem cells’ bioenergetic metabolism is intrinsically regulated by pluripotency factors and in turn metabolites can regulate some epigenetic machinery that may affect the state of pluripotency (Tsogtbaatar et al., 2020). Consequently, in order for hAFSCs from fetus-affected gestations to be successfully applied in clinical practice, first, the state of their metabolism must be elucidated. In addition, for the neuro-therapeutic approach, hAFSC neurogenic potential should also be evaluated.

Therefore, in this study, we compared the bioenergetic/metabolic and neurogenic characteristics of hAFSCs, obtained from normal and fetus-affected gestations. Results of our analysis demonstrated that hAFSCs from polyhydramnios samples were exploiting oxidative phosphorylation approximately twice as efficiently as hAFSCs from normal gestations (Figure 2A). In accordance to OXPHOS, hAFSCs from polyhydramnios were noticed to have higher levels of intracellular ATP (Figure 2C), as well as higher expression of *NRF1* gene (Figure 2G), which is known as one of the main mitochondrial respiratory function regulators (Yuan et al., 2018). Not surprisingly, mitochondrial membrane potential was registered to be higher in hAFSCs from normal gestations in comparison to hAFSCs from polyhydramnios (Figure 2B), as it is widely accepted that mitochondrial membrane potential drops in the state of active respiration (Nicholls, 2004). In addition, our study demonstrated that hAFSCs from healthy pregnancies were significantly more prone to neurogenic differentiation induction compared to hAFSCs from polyhydramnios (Figure 3). Furthermore, hAFSCs from normal pregnancies were more positive for stemness markers, such as CD73, CD90, and CD105 (Figure 1D). Aforementioned differences could possibly be explained by considering the gestational age of investigated cells, as in our study, hAFSCs were obtained from 16 to 17 weeks of normal gestations, whereas hAFSCs from polyhydramnios were obtained at 32 weeks. Although some studies, also including our previous research, showed that hAFSCs may be comparably potent in their ability to differentiate to certain lineages despite their differences in gestational age (Hamid et al., 2017; Gasiūnienė et al., 2019b; Zentelytė et al., 2020), other studies contradict this notion (Shaw et al., 2017; Huang et al., 2020). Furthermore, it is argued that pluripotent SCs typically have a lower rate of OXPHOS and maintain higher mitochondrial membrane potential in comparison to more differentiated SCs (Tsogtbaatar et al., 2020). In addition, research with mouse embryonic SCs (Schieke et al., 2008) revealed that SCs with high mitochondrial membrane potential may differentiate into all three germ layers, whereas SCs with lower mitochondrial membrane potential are restricted only to the mesodermal lineage. Therefore, results obtained in our study would suggest that hAFSCs from polyhydramnios samples have lower neurogenic differentiation potential due to the gestational age-determined changes in metabolic status.

On the other hand, it should be emphasized that hAFSCs from normal and pathological pregnancies were comparable in glycolysis rate (Figure 2A), as well as gene and protein expression of certain pluripotency-associated factors, which are known to directly regulate glycolysis in pluripotent SCs, such as LIN28A (Figures 1B, 8A; Tsogtbaatar et al., 2020). However, gene expression of other pluripotency-associated factors that are involved in glycolysis regulation of *OCT4* and *MYC* was significantly higher in hAFSCs from polyhydramnios samples compared to hAFSCs from normal gestations (Figure 1B). It should be stressed out that transcription factor OCT4 is known for the direct modulation of pyruvate kinase M2 (*PKM*) gene expression (Kim et al., 2015). In addition, it was also demonstrated to upregulate *GLUT1* expression *via* binding to the *GLUT1* enhancer site (Yu et al., 2019). Similarly, c-MYC regulates transcription of *PKM* and *LDHA* (Cao et al., 2015), which in our study was also more pronounced in hAFSCs from polyhydramnios (Figure 2G). On the other hand, c-MYC itself is involved in transcriptional regulation of certain stem cell marker proteins, such as SOX2 and OCT4 (Sisodiya et al., 2012). Moreover, c-MYC was shown to upregulate expression of TNFα (Liu et al., 2015), which in our study was also significantly higher expressed and secreted by hAFSCs from polyhydramnios samples (Figures 8F,G). Therefore, at this stage, the exact role of c-MYC in hAFSCs obtained from normal and fetus-affected gestations remains unclear and further more thorough investigations are needed. However, the cross-examination, involving hAFSCs from healthy gestations obtained at 32 weeks, is not eligible or ethical. Consequently, it is impossible to decipher the precise role of gestational age in the metabolic characteristics of tested cells.

Regarding neurogenic differentiation induction in hAFSCs from healthy gestations, the most effective results were achieved when using the combination of 50 ng/ml BDNF + 100 ng/ml NGF + 2 μM RA + 5 mM KCl (Protocol No. II; Figures 5A,B, 6B, 7B,C). For example, the highest expression of neural differentiation-associated genes *NCAM*2, *NES*, *MAP2*, *VEGFA*, *BDNF*, and *NTF4* was registered upon neurogenic induction with the aforementioned chemical cocktail. However, the most characteristic neuro-like morphology was observed when this treatment was supplemented with 1 mM 8-Bromo-cAMP and 0.3 mM IBMX (Protocol No. III; Figure 3A). Our results coincide with Bonaventura et al. (2015) (Bonaventura et al., 2015), who demonstrated that a similar chemical cocktail (1 mM dbcAMP + 0.5 mM IBMX + 20 ng/ml EGF + 40 ng/ml bFGF + 10 ng/ml NGF + 10 ng/ml BDNF) was potent in hAFSC neurogenic differentiation induction. However, the chemical combination consisting only of 1 mM 8-Bromo-cAMP, 0.3 mM IBMX, 2 μM RA, and 5 mM KCl (protocol No. I) was the least efficient, while treatment with 50 ng/ml BDNF + 100 ng/ml NGF + 2 μM RA + 5 mM KCl proved to be the most effective in neurotrophic factors’ gene induction (Figures 7B,C). For this reason, we further tested the secretome of hAFSCs upon neural differentiation induction with this particular chemical combination. Recent studies by other authors (Kukumberg et al., 2021) showed that hAFSCs under hypoxic conditions, in comparison to normoxic state, secrete larger quantities of growth factors such as BDNF and VEGF. Results of our ELISA analysis revealed that secretion of BDNF and VEGF proteins is also increased after the induction of neurogenic differentiation by a comparable degree in hAFSCs from both normal and pathological pregnancies (Figures 8C,D). In addition, upon neurogenic differentiation induction in hAFSCs, the intracellular levels of neuronal differentiation-associated proteins Nestin and LIN28A were upregulated more strongly in cells from polyhydramnios samples in comparison to cells obtained from healthy gestations. These observations may suggest that hAFSCs obtained from polyhydramnios should also be tested further for their neurotrophic potential and possible clinical applicability.

Surprisingly, in hAFSCs from polyhydramnios samples, secretion of pro-inflammatory cytokine TNFα was also detected (Figure 8F). However, upon neurogenic differentiation, it was significantly reduced. We wondered if secretion of TNFα by hAFSCs obtained from polyhydramnios samples may be associated with the intensity of ROS generation within these cells. Results of cellular ROS analysis revealed that hAFSCs from polyhydramnios samples indeed produce higher quantities of intrinsic ROS (Figures 2D–F). In addition, *TNFA* gene expression was also significantly higher in hAFSCs from pathological gestations (Figure 8F). Previous research by other authors (Kim et al., 2012) demonstrated that increased CD13 (aminopeptidase N) expression reduces ROS generation in cancer stem cells. In our study, such a negative correlation between the expression of CD13 (Figure 1C) and ROS production was also evident in hAFSCs. We believe that all these observations could possibly be explained by the inflammatory state of polyhydramnios itself, as treatment with the anti-inflammatory agents is suggested for the reduction of amniotic fluid volumes (Hamza et al., 2013). However, further research is needed in order to explain the role and molecular pathways of TNFα and ROS in the biology of hAFSCs obtained from polyhydramnios.

Nevertheless, subsequent analysis of inflammation-associated cytokines (IL-1β, IL-6, and IL-10) revealed that hAFSCs from polyhydramnios samples do not differ from healthy fetuses-derived hAFSCs in secretion of these cytokines, as no secretion of IL-1β and IL-10 was detected at all and levels of secreted IL-6 were comparable between tested groups (Figure 8E). Although IL-6 is generally regarded as a major inducer of immune and inflammatory cascades, accumulating data emphasize its role within the central nervous system as well, as IL-6 promotes differentiation of neural stem cells into glutamate-responsive neurons and several astroglia cell types (Islam et al., 2009). In addition, IL-6 was also recently described to have an anti-inflammatory activity, as its effect depends on concentration and combination with other pro-inflammatory cytokines (Borsini et al., 2020). As it is evident from the results of our research, IL-6 secretion increases upon neural differentiation induction in hAFSCs from the Pathology group, whereas in hAFSCs from normal gestations, secretion of IL-6 slightly diminishes, although these differences are not statistically significant. Obviously, more comprehensive examination of hAFSC secretome would be necessary in order to refine the whole picture of paracrine activities of these cells. Recent research performed by Costa et al. (2021) showed that fetal hAFSCs (from the second trimester of gestations) and perinatal hAFSCs (obtained during scheduled C-sections) do differ in cytokine and chemokine profile. For example, several proteins, such as extracellular matrix metalloproteinase inducer (EMMPRIN) and interleukin 8 (IL-8), were found to be exclusively enriched in fetal but not in perinatal hAFSCs upon hypoxic preconditioning. In the aforementioned study, BDNF protein was enriched in all hypoxia-induced extracellular vesicles of hAFSCs regardless of gestational age, similarly to our study, where the level of BDNF secretion was comparable in both “Normal” and “Pathology” groups. Furthermore, recent research by Castelli et al. (2021) revealed that the secretome of hAFSCs was able to activate pro-survival and anti-apoptotic pathways and exhibited neuro-protective activity in an ischemia/reperfusion SH-SY5Y cell model, while activating intracellular BDNF signaling.

To sum up, results of our research expand the knowledge about the general characteristics and neurogenic potential of hAFSCs obtained from healthy and fetus-affected gestations. In this study, we demonstrated that hAFSCs from healthy gestations and gestations with fetus pathology concomitant with polyhydramnios do differ in their metabolic status, as hAFSCs from polyhydramnios samples rely on oxidative phosphorylation more heavily and are more energetic compared to hAFSCs from healthy fetuses. Although hAFSCs from normal gestations were shown to have stronger neurogenic potential, hAFSCs from these two different sources are comparable in neurotrophic factor secretion, in both control (untreated) and neuro-induced states. However, different patterns of pro-inflammatory cytokine TNFα secretion were characteristic for tested hAFSC groups, as hAFSCs from polyhydramnios samples were producing extracellular TNFα, whereas no secretion of TNFα was detected from hAFSCs, obtained from healthy gestations. Therefore, in future studies, *in vivo* potential as well as strategies that could improve the characteristics of hAFSCs derived from diseased fetuses should be investigated in order for those cells to be successfully applied for regenerative medicine purposes, particularly for infants, either in the prenatal or neonatal stage.

## Limitations of This Study

It should be emphasized that sample groups in this study were quite small (three samples per group). Groups of studied hAFSCs were also different in gestational age (cells from healthy pregnancies were obtained at 16–17 weeks of gestation, whereas cells from polyhydramnios were obtained at 32 weeks of gestation). As we have already stated, it is impossible to obtain hAFSCs from healthy pregnancies at 32 weeks of gestation in the clinical practice, as such interventions have no clinical utility and are not ethical. In addition, hAFSCs from gestations with polyhydramnios may also be obtained only when polyhydramnios condition occurs (in most cases, polyhydramnios develops late in the second or in the third trimester of pregnancy). Therefore, this particular shortcoming of our study is determined by the settings in the clinical practice and pathophysiology of polyhydramnios itself. The heterogeneity in diagnoses in the “Pathology” group samples is also a limitation of this study. However, this group was uniform for gestational age and concomitant polyhydramnios state.

## Data Availability Statement

The raw data supporting the conclusions of this article will be made available by the authors, without undue reservation.

## Ethics Statement

The studies involving human participants were reviewed and approved by Vilnius Regional Biomedical Research Ethics Committee, No. 158200-18/7-1049-550, version No. 1. The patients/participants provided their written informed consent to participate in this study.

## Author Contributions

GV conceived the idea and research questions, performed a formal analysis, investigation, and visualization, supervised the ongoing study, and prepared the first complete draft of the manuscript. AZ performed a formal analysis, investigation, and visualization, and aided in manuscript preparation and editing. EB performed investigation and formal analysis. RN reviewed and edited the manuscript. All authors approved the final manuscript.

## Conflict of Interest

The authors declare that the research was conducted in the absence of any commercial or financial relationships that could be construed as a potential conflict of interest.

## Footnotes

1
http://www.doubling-time.com/compute.php
