# Supplementary material for: Metabolic Profile and Neurogenic Potential of Human Amniotic Fluid Stem Cells From Normal vs. Fetus-Affected Gestations
Source: Front Cell Dev Biol. 2021 Jul 16;9:700634. doi: 10.3389/fcell.2021.700634 (PMC8322743; doi:10.3389/fcell.2021.700634)
Supplement: Supplementary file 1 [file Data_Sheet_1.PDF]

Metabolic profile and neurogenic potential of human amniotic fluid stem cells from normal vs. fetus affected gestations

Giedrė Valiulienė[1*], Aistė Zentelytė[1], Elizabet Beržanskytė[1], Rūta Navakauskienė[1]

[1]Department of Molecular Cell Biology, Institute of Biochemistry, Life Sciences Center, Vilnius University, Saulėtekio av. 7, LT-01257 Vilnius, Lithuania.

*corresponding author: giedre.valiuliene@bchi.vu.lt

Table S1. Antibodies used for flow cytometry analysis

Antibody	Fluorophore	Manufacturer
Mouse izotype control IgG1	PE	Biolegend
Anti-CD105		
Anti-CD166		
Mouse izotype control IgG1	Alexa Fluor® 488	
Mouse izotype control IgG2a		
Anti-CD31		
Anti-HLA-ABC		
Anti-HLA-DR		
Mouse izotype control IgG2a	FITC	
Anti-CD34		
Mouse izotype control IgG1	APC	
Mouse izotype control IgG2b		
Anti-CD44		
Anti-CD56		
Anti-CD133		
Anti-CD146		
Anti-CD309		
Anti-CD338		
Anti-LIN28a	-	
Mouse izotype control IgGM	PE	Exbio
Anti-CD15		
Mouse izotype control IgG1	FITC	
Anti-CD9		
Anti-CD73		
Mouse izotype control IgG1	APC	
Anti-CD13		
Anti-CD90		
Anti-CD117		
Rabbit izotype control IgG	FITC	Abcam
Anti-TUBB3		
Anti-Nestin	-	Novus Biologicals
Anti-Musashi1		

Table S2. List of primers used for gene expression analysis

No.	Name	Primer sequence
1	*BDNF*	F: TAACGGCGGCAGACAAAAAGA<br>R: TGCACTTGGTCTCGTAGAAGTAT
2	*CAT1*	F: TGGAGCTGGTAACCCAGTAGG<br>R: CCTTTGCCTTGGAGTATTTGGTA
3	*ERRA*	F: AGGGTTCCTCGGAGACAGAG<br>R: TCACAGGATGCCACACCATAG
4	*FGFR1*	F: AGAAGAGCGACCCTCACATCA<br>R: CGGTTAGCACACACTCCTTTG
5	*GAD1*	F: GCGGACCCCAATACCACTAAC<br>R: CACAAGGCGACTCTTCTCTTC
6	*GAPDH*	F: AGTCCCTGCCACACTCAG<br>R: TACTTTATTGATGGTACATGACAAGG
7	*GFAP*	F: GGCAAAAGCACCAAAGACGG<br>R: GGCGGCGTTCCATTTACAAT
8	*GPX1*	F: CAGTCGGTGTATGCCTTCTCG<br>R: GAGGGACGCCACATTCTCG
9	*HBEGF*	F: ATCGTGGGGCTTCTCATGTTT<br>R: TTAGTCATGCCCAACTTCACTTT
10	*HIF1A*	F: CCAACAGTAACCAACCTCAG<br>R: TCCTGTGGTGACTTGTCCTT
11	*LDHA*	F: ATGGCAACTCTAAAGGATCAGC<br>R: CCAACCCCAACAACTGTAATCT
12	*LIN28A*	F: TTGTCTTCTACCCTGCCCTCT<br>R: GAACAAGGGATGGAGGGTTTT
13	*MAP2*	F: CCAATGGATTCCCATACAGG<br>R: TCCTTGCAGACACCTCCTCT
14	*MYC*	F: CAGCGACTCTGAGGAGGAAC<br>R: GCTGTGAGGAGGTTTGCTGT
15	*NANOG*	F: AGATGCCTCACACGGAGACT<br>R: GTTTGCCTTTGGGACTGGTG
16	*NCAM1*	F: TGTCCGATTCATAGTCCTGTCC<br>R: CTCACAGCGATAAGTGCCCTC
17	*NCAM2*	F: AGGAAGGTGTTAGGTCACGGT<br>R: CCTTTGGCATCTGTTGCTTGA
18	*NES*	F: CTGCTACCCTTGAGACACCTG<br>R: GGGCTCTGATCTCTGCATCTAC
19	*NEUROD1*	F: ATGACCAAATCGTACAGCGAG<br>R: GTTCATGGCTTCGAGGTCGT
20	*NGF*	F: TGTGGGTTGGGGATAAGACCA<br>R: GCTGTCAACGGGATTTGGGT
21	*NOTCH1*	F: GGTGAGACCTGCCTGAATG<br>R: GTTGGGGTCCTGGCATC
22	*NRF1*	F: AGGCTGGGGGAAAGAAAG<br>R: CCAACCTGGATAAGTGAGAC
23	*NSE*	F: CCCACTGATCCTTCCCGATACAT<br>R: CCGATCTGGTTGACCTTGAGCA

No.	Name	Primer sequence
24	*NTF3*	F: CCGTGGCATCCAAGGTAACAA<br>R: GCAGTTCGGTGTCCATTGC
25	*NTF4*	F: GCAAGGCTGATAACGCTGAG<br>R: CAATGCCCGCACATAGGACT
26	*NTRK1*	F: CCCCATCCCTGACACTAACA<br>R: GCACAAGGAGCAGCGTAGAA
27	*NTRK2*	F: TCGTGGCATTTCCGAGATTGG<br>R: TCGTCAGTTTGTTTCGGGTAAA
28	*NTRK3*	F: GCCAGTATCAACATCACGGAC<br>R: AGCCGGTTACTTGACAGGTTT
29	*OCT4*	F: CGAGAAGGATGTGGTCCGAG<br>R: CAGAGGAAAGGACACTGGTC
30	*PDGFRA*	F: TTGAAGGCAGGCACATTTACA<br>R: GCGACAAGGTATAATGGCAGAAT
31	*PDK1*	F: GAGAGCCACTATGGAACACCA<br>R: GGAGGTCTCAACACGAGGT
32	*PKM*	F: ATGTCGAAGCCCCATAGTGAA<br>R: TGGGTGGTGAATCAATGTCCA
33	*PPARG1CA*	F: GCAATTGAAGAGCGCCGTGTGA<br>R: CTGTCTCCATCATCCCGCAGAT
34	*RPL13A*	F: GTTGATGCCTTCACAGCGTA<br>R: AGATGGCGGAGGTGCAG
35	*SOD2*	F: CGTGACTTTGGTTCCTTTGAC<br>R: ATTTGTAAGTGTCCCCGTTCC
36	*SOX2*	F: TGGACAGTTACGCGCACAT<br>R: CGAGTAGGACATGCTGTAGGT
37	*SYP*	F: CTCGGCTTTGTGAAGGTGCT<br>R: CTGAGGTCACTCTCGGTCTTG
38	*S100B*	F: ATGTCTGAGCTGGAGAAGG<br>R: CTCATGTTCAAAGAACTCGTG
39	*TGFB1*	F: CAATTCCTGGCGATACCTCAG<br>R: GCACAACTCCGGTGACATCAA
40	*TNFA*	F: CCTCTCTCTAATCAGCCCTCTG<br>R: GAGGACCTGGGAGTAGATGAG
41	*TNFR1*	F: TCACCGCTTCAGAAAACCACC<br>R: GGTCCACTGTGCAAGAAGAGA
42	*TNFR2*	F: TTCATCCACGGATATTTGCAGG<br>R: GCTGGGGTAAGTGTACTGCC
43	*TPH1*	F: ACGTCGAAAGTATTTTGCGG<br>R: ACGGTTCCCCAGGTCTTAATC
44	*TPH2*	F: CAAAAATGACGACAAAGGCAACA<br>R: CCTCAGTGCTTTTACCAATCCA
45	*TUBB3*	F: CTCAGGGGCCTTTGGACATC<br>R: CAGGCAGTCGCAGTTTTCAC
46	*VEGFA*	F: AGGGCAGAATCATCACGAAGT<br>R: AGGGTCTCGATTGGATGGCA
47	*VIM*	F: TCTCTGAGGCTGCCAACCG<br>R: CGAAGGTGACGAGCCATTTCC
